# The role of cinnamon as a modulator of the expression of genes related to antioxidant activity and lipid metabolism of laying quails

**DOI:** 10.1371/journal.pone.0189619

**Published:** 2017-12-21

**Authors:** Marisa Silva Bastos, Ana Paula Del Vesco, Thaís Pacheco Santana, Thailine Santana Santos, Gregório Murilo de Oliveira Junior, Roberta Pereira Miranda Fernandes, Leandro Teixeira Barbosa, Eliane Gasparino

**Affiliations:** 1 Animal Science Department, Federal University of Sergipe, São Cristóvão s/n, Brazil; 2 Physiology Departament, Federal University of Sergipe, São Cristóvão s/n, Brazil; 3 Animal Science Department, Estadual University of Maringá, Maringá, Brazil; Leibniz-Institut fur Pflanzengenetik und Kulturpflanzenforschung Gatersleben, GERMANY

## Abstract

Since cinnamon has vitamins and minerals in addition to antioxidants compounds in its chemical composition studies have shown the potential of cinnamon supplementation on some important characteristics in the performance of birds. Thus, this study was conducted under the hypothesis that the inclusion of cinnamon in the laying quail diet could influence the performance of the birds through the expression of genes related to antioxidant activity and lipid metabolism. To test this hypothesis, 144 Japanese quail (*Coturnix japonica*) with an initial age of 18 weeks and average weight of 133g were distributed in a completely randomized design with two treatments: no cinnamon supplementation (NCS—control group) and with supplementation of 9g/kg of cinnamon powder (CPS). The experiment lasted for 84 days. At the end of the experimental period, six animals from each treatment were euthanized by cervical dislocation, blood was collected and organs weighed. Liver tissue was collected for gene expression and biochemical analyses. We observed a significant effect of cinnamon inclusion on the weight of the pancreas (P = 0.0418), intestine (P = 0.0209) and ovary (P = 0.0389). Lower weights of the pancreas and intestine, and a higher ovary weight was observed in birds receiving the CPS diet. Quails fed with cinnamon supplementation also had better feed conversion per egg mass (2.426 g /g, P = 0.0126), and higher triglyceride (1516.60 mg/dL, P = 0.0207), uric acid (7.40 mg/dL, P = 0.0003) and VLDL (300.40 mg/dL, P = 0.0252) contents. A decreased content of thiobarbituric acid reactive substances (TBARS) and lower catalase activity was observed in the liver of quails from the CPS diet (0.086 nmoles/mg PTN, and 2.304 H_2_O_2_/min/mg PTN, respectively). Quails from the CPS group presented significantly greater expression of *FAS* (fatty acid synthase, 36,03 AU), *ACC* (Acetyl-CoA Carboxylase, 31.33 AU), *APOAI* (apolipoprotein A-I, 803,9 AU), *ESR2 (*estrogen receptor 2, 0.73 AU) *SOD* (superoxide dismutase, 4,933.9 AU) and *GPx7* (glutathione peroxidase 7, 9.756 AU) than quails from the control group. These results allow us to suggest that cinnamon powder supplementation in the diet of laying quails can promote balance in the metabolism and better performance through the modulation of antioxidant activity and the expression of genes related to lipid metabolism.

## Introduction

The lipid metabolism in laying birds plays an important role in egg formation since most of the yolk precursors are synthesized in the liver and transported to the follicle in the form of very low density lipoprotein (VLDL). The continuous egg production can overload the metabolism, causing disturbances that block the transport of VLDL, thus accumulating triglycerides in the liver [1,2].

The intense metabolism related to continuous egg production may also be related to the higher production of reactive oxygen species (ROS) [3]. These ROS act by damaging the cellular components of tissues, mainly in the liver, causing cell death and the loss of liver function, leading to bleeding and serious infections [1, 4–5]. According to Nadia et al. [6], the action of free radicals can decrease the fertility of the birds if the tissues have no efficient antioxidant mechanisms in the reproductive system. Thus, one alternative is supplementation with compounds that enhance the body's antioxidant defense, such as those found in medicinal herbs.

Some herbs have been studied as potential antioxidants because they have the ability to combat ROS. An example of this is cinnamon (*Cinnamomum zeylanicum L*.), an arboreal plant of Asian origin, which belongs to the family of lauraceae, and is widely used in the food and cosmetics industry. Cinnamon has vitamins and minerals antioxidants in its chemical composition in addition to essential oil component [7]. According to Sivapriya [8], the essential oil component contains cinnamaldehyde as a major constituent with its derivatives cinnamic acid, cinnamyl alcohol, ethyl cinnamate, cinnamyl acetate and 2-methoxy cinnamaldehyde. The oil component also contains eugenol, linalool, coumarin, carvone, carvacrol and β-caryophyllene. Cinnamon bark contains procyanidins and catechins that also possess antioxidant activities [9].

Phenolic compounds such as eugenol have been shown as efficient at combating free radicals (ROS) because of their powerful antioxidant activity and radical-scavenging activity; the chemical structure of eugenol determines its antioxidant activity, providing a substrate for reaction with free radicals, and inhibiting lipid peroxidation [10]. In addition, eugenol may also influence the enzyme activity of the glutathione system, which is considered one of the main antioxidant defense systems [11].

Studies have shown the potential of cinnamon supplementation on some important characteristics in the performance of birds. Şimşek [12] observed that cinnamon oil exhibited significant antioxidant activity in Japanese quail, mainly under thermal stress conditions; the author related this result to the high content of phenolic compounds present in cinnamon. Asides cinnamon oil, supplementation with cinnamon powder can also increase meet quality related to the antioxidant activity [13].

Despite the available information, studies showing the action of cinnamon powder on the performance of laying hens are scarce, so this study was developed under the hypothesis that the inclusion of powdered cinnamon in a laying quail diet could influence the performance of birds through the expression of genes related to antioxidant activity and to lipid metabolism. To test this hypothesis, the objective of this study was to evaluate the productive performance, blood parameters, antioxidant activity in the hepatic tissue, oxidative stress markers, and the expression of acetyl-CoA carboxylase (*ACC*), fatty acid synthase (*FAS*), apolipoprotein AI (*APOA-I*), apolipoprotein B (*APOB*), superoxide dismutase (*SOD*) and glutathione peroxidase 7 (*GPx7*) in the liver of Japanese quails fed diets supplemented with cinnamon powder or without cinnamon supplementation.

## Materials and methods

This experiment was approved by the animal production research ethics committee of the Federal University of Sergipe (Protocol N^o.^ 09/2015).

### Animals and experimental design

One hundred forty-four Japanese quail (*Coturnix japonica*) obtained from a commercial hatchery (VICAMI, Assis, São Paulo, Brazil) were used in this experiment. The quails aged 15 weeks and with an average weight of 133g were housed in a masonry shed, uniformly distributed in laying cages (0.50 x 0.15 x 0.35 m).

For the standardization of egg production, a daily monitoring of the production was performed for a period of three weeks. The experiment was then started when the experimental units presented a posture rate of about 85% and 18 weeks of age. The birds were distributed in a completely randomized design with two treatments: no cinnamon supplementation (NCS—control group) and with cinnamon powder supplementation of 9g/kg (CPS) to replace the inert filler (Kaolin). Each treatment consisted of six replicates with 12 birds each, totaling 72 quails per treatment. The supplementation level was chosen according to the results of literature reference [14].

The experimental diets (Table 1) were formulated based on corn and soybean meal according to the nutritional recommendations found in the Brazilian Tables for Poultry and Swine [15]. The experiment lasted for 84 days. To avoid damages in the diets during the experimental period, the diets were prepared every 15 days. The birds received water and feed *ad libitum* and a 16-hour daily light program was used.

**Table 1:** Percent composition and nutritional values of basal diet.

Diet	(%)
Corn	57.792
Soybean meal 45%	30.450
Soy oil	1.561
Dicalcium phosphate	1.091
Limestone	6.802
Salt	0.323
L-Lysine HCl	0.261
DL-Methionine	0.396
L-Threonine	0.024
Vitamin and mineral mixture^1^	0.100
Kaolin (Inert filler)	1.200
Total	100.000
**Nutritional composition**	
Metabolizable energy (kcal/kg)	2807
Crude protein (%)	18.80
Fat (%)	4.101
Calcium (%)	2.922
Available phosphorus (%)	0.304
Sodim (%)	0.146
Chlorine (%)	0.242
Potassium (%)	0.725
**Digestible amino acids** (%)	
Methionine	0.642
Methionine + Cysteine	0.900
Lysine	1.097
Threonine	0.658
Tryptophan	0.206

^1^Vitamin and mineral misture (guaranteed levels per kg of product): Ac. Folic (min.) 200mg; ac. pantothenic (min.) 5.350 mg; copper (mín.) 4.000 mg; iron (mín) 20 g; iodine (mín.) 1.500 mg; manganese (mín.) 75 g; niacin (mín.) 19,9 g; selenium (mín.) 250 mg; Vit. A (mín.) 8.000.000 IU; Vit. B12 (mín.) 10.000 mcg; Vit. B2 (mín.) 4.000 mg; Vit. B6 (mín.) 1.000 mg; Vit. D3 (mín) 2.000.000 IU; Vit. E (mín.) 15.000 IU; Vit. K3 (mín.) 2.000 mg; zinc (mín.) 50 g.

### Eggs production

The eggs were collected daily, stored in boxes identified by experimental unit, counted and weighed. All broken eggs and those without shells were also counted. Weekly, to record the feed consumption, the amount of feed provided in a seven-day period was weighed, as was the remaining diet at the end of each week.

After the daily collection, all the eggs were weighed in a digital balance (0.01 g) and the average weight was obtained by dividing the egg weight of each cage by the number of eggs. Data for egg mass (g/bird/day) were obtained by multiplying the number of eggs produced in each cage by the average egg weight. The feed conversion by egg mass (g/bird/day) was calculated by dividing feed intake (g) by the mass of eggs (g) produced during the experimental period.

At the end of the experimental period, six eggs with an average weight of the replicate were separated for the following analyses: yolk weight (g), shell weight (g), and albumen weight (g). Using these data, the percentages of yolk, shell and albumen were calculated.

The shells, which were numbered, were placed at room temperature for a 24 hours to dry. After drying, they were weighed.

From the values of egg weight, yolk weight and shell weight, the weight of the albumen was determined and the percentages of each part of the egg were calculated.

### Relative weights and blood parameters

At the end of the proposed experimental period of 84 days, six animals from each treatment were weighed and euthanized by cervical dislocation. These animals were eviscerated to evaluate the relative weights of the organs. Weights of the liver, pancreas, intestine, ovary, gizzard, and abdominal fat were measured, along with intestine length. The relative weight was calculated as (organ weight/bird weight) x100.

To perform the enzymatic activity analysis of creatine kinase (CK), aspartate aminotransferase (AST), and alanine aminotransferase (ALT), and the content of total cholesterol, creatinine, glucose, triglycerides, uric acid and very low density lipoprotein (VLDL), blood samples were collected from six birds per treatment, collected from the jugular vein, in collector tubes with EDTA and kept on ice. The blood samples were centrifuged (1500 x g, 10 min, 4°C), and the plasma was collected and stored at -20°C until analysis.

The creatinine, glucose, triglycerides, uric acid, cholesterol, and VLDL contents, as well as the ALT, AST and CK activity analyses, were performed according to colorimetric methods with the following kits: CREATININE-PP-MS 80022230066, GLUCOSE- PP-MS 80022230067, TRIGLYCERIDES-PP-MS 80022230062, URIC ACID-PP-MS 80022230065, CHOLESTEROL HDL-PP-MS 800222300068, ALT-MS 80022230086, AST-MS80022230083 and CK-NAC-PP-MS 80022230088, respectively, according to the manufacturer's recommendations (Gold Analisa, Belo Horizonte, Minas Gerais, Brazil).

### Catalase activity and TBARS

For the biochemical evaluations, part of the liver of five animals from each treatment was collected and stored in liquid nitrogen until the analysis.

The determination of TBARS concentration was based on the ability of thiobarbituric acid to bind to oxidized lipids. For this, 100mg of the tissue was weighed and 1ml of phosphate buffer was added (0.1M, pH 7.4). The homogenate was centrifuged at 4°C for 10 minutes at 10,000 x g. Then, 500μl of the supernatant was collected and 250μl of 28% TCA diluted in 0.25N HCl, 250μl of 1% thiobarbituric acid diluted in acetic acid (1:1) and 125μl of 5mM BHT (butylated hydroxy toluene) was added. The solution was homogenized and heated in a water bath for 15 minutes at 95°C. It was then centrifuged at 4°C for 10 minutes at 10,000 x g. Then, the supernatant was analyzed in a spectrophotometer at 535 nm. The concentration of TBARS was determined using the molar extinction coefficient ε = 1.56 x 10^5^ L.mol^-1^.cm ^-1^, according to Lambert Beer's law. Usually this concentration is represented in nmoles per mg of protein.

The analysis of catalase activity was based on the ability of the CAT enzyme present in the samples to convert hydrogen peroxide (H_2_O_2_) to water and molecular oxygen, as described by Aebi [16]. For this, 100mg of the tissue was weighed and added to 1ml of phosphate buffer (0.1M, pH 7.2). The homogenate was centrifuged at 4°C for 10 minutes at 10,000 x g. In a clean tube, 50μL of phosphate buffer (pH 7.2) and 40μL of distilled water were added. This mixture was kept in a water bath at 30°C for one minute, and then 10μL of the sample supernatant and 900μL of H_2_O_2_ (10mM) were added, before the solution was homogenized. The absorbance was then measured in a spectrophotometer at 240 nm, after being blanked with H_2_O_2_ (10 mM); these measurements were read every minute for 5 minutes. It is known that 1U of catalase is able to hydrolyze 1μmol of H_2_O_2_ per minute (ε = 39.4 L.mol^-1^cm^-1^). The absorbance used in this expression was the delta obtained from the five absorbance values read (final absorbance—initial absorbance/4).

To adjust the enzymatic catalase assays and TBARS content, the quantification of total proteins was performed by the Bradford method [17].

### Gene expression

For the gene expression analysis (bird was the experimental unit, n = 4), liver samples were collected and stored in RNA Holder (BioAgency Biotecnologia, São Paulo, Brasil) at -20°C until total RNA was extracted.

Total RNA was extracted using Trizol® (Invitrogen, Carlsbad CA, USA) according to the manufacturer’s instructions (1 mL per 100 mg of tissue). All of the materials used were pre-treated with the RNase inhibitor RNase AWAY® (Invitrogen, Carlsbad, CA, USA). The total RNA concentration was measured using a spectrophotometer at a wavelength of 260 nm. RNA integrity was analyzed by 1% agarose gel electrophoresis and 10% ethidium bromide staining, and visualized under ultraviolet light. The RNA samples were treated with DNase I (Invitrogen, Carlsbad, CA, USA) according to the manufacturer’s instructions to remove potential genomic DNA contamination.

A SuperScript^TM^ III First-Strand Synthesis Super Mix (Invitrogen Corporation, Brasil) kit was used for cDNA synthesis from 1 μg of DNase-treated total RNA, according to the manufacturer’s instructions. The cDNA concentration was measured using a spectrophotometer at a wavelength of 260 nm. The cDNA samples were diluted to 40 ng/μL and stored at -20°C until further use as a template in the amplification reaction. Real-time PCR reactions were performed using the fluorescent dye SYBR GREEN (SYBR® GREEN PCR Master Mix, Applied Biosystems, USA). The amplification reaction consisted of 5 μL of diluted cDNA, 0.5 μL of each primer (forward and reverse) at 10 μM (final concentration: 200 nM), 12.5 μL of SYBR® GREEN PCR Master Mix, and water to a total volume of 25 μL. To measure the efficiency of each primer/gene set, a series of 25 μL reactions was analyzed as described above using 5 μL of a serial dilution of pooled cDNA as the template. The thermal cycling parameters for all genes were as follows: hot-start at 95°C for 10 min, followed by 40 cycles of denaturation at 95°C for 15 s and annealing/extension at 60°C for 1 min, and ending with a melt curve from 65–95°C.

The primers used for Acetyl-CoA Carboxylase (*ACC*) and fatty acid synthase (*FAS*) were designed according to Lei and Lixian [18]; primers used for apolipoprotein A-I (*APOAI)* and apolipoprotein B (*APOB*) were designed according to Jiang et al. [19], and primers used for the superoxide dismutase (*SOD*), glutathione peroxidase 7 (*GPx7*), and estrogen receptor 2 (*ESR2)* reactions were designed based on the gene sequences deposited at www.ncbi.nlm.nih.gov (accession numbers, NM_205064.1, NM_001163245.1 e XM_015865463.1, respectively) using the site www.idtdna.com. Two endogenous controls, *ß-actin* and *GAPDH*, were tested, and *ß-actin* (accession number L08165) was selected because its amplification efficiency was higher and similar to the amplification efficiency of the target genes (Table 2). All of the analyses were performed in duplicate.

**Table 2:** Primers for qRT-PCR.

Genes	Amplicon (Bp)	TA (°C)	Sequences of primers (5’-3’)^1^
*ACC*^*2*^	136	60	AATGGCAGCTTTGGAGGTGT TCTGTTTGGGTGGGAGGTG
*FAZ*	107	60	CTATCGACACAGCCTGCTCCT CAGAATGTTGACCCCTCCTACC
*APOA-I*	217	60	GTGACCCTCGCTGTGCTCTT CACTCAGCGTGTCCAGGTTGT
*APOB*	196	60	GACTTGGTTACACGCCTCA TAACTTGCCTGTTATGCTC
*ß-actina*	136	60	ACCCCAAAGCCAACAGA CCAGAGTCCATCACAATACC
*SOD*	126	60	TGGACCTCGTTTAGCTTGTG ACACGGAAGAGCAAGTACAG
*GPx7*	140	60	TTGTAAACATCAGGGGCAAA TGGGCCAAGATCTTTCTGTAA

^1^Bp, base pairs; TA, temperature of anelamento

^2^*ACC*, Acetyl-CoA-carboxylase; *FAS*, Fatty acid synthase; *APOA-I*, Apolipoprotein A-I; *APOB*, Apolipoprotein B; *SOD*, Superoxide dismutase; *GPX7*, Glutathione peroxidase 7.

The amplification efficiencies (90% to 110%) were similar for the genes of interest (Table 2). Analysis of the dissociation curves did not reveal any non-specific PCR products, such as the formation of primer dimers, thus demonstrating the reliability of the data for estimating the mRNA expression of the evaluated genes. The endogenous control, *β-actin*, did not show any significant differences between treatments, which confirmed its suitability as a control.

### Statistical analysis

The Shapiro-Wilk test was used to verify the normality of the expression data of the genes under study (expressed as 2-^ΔCt^) and the other data evaluated. Student’s t test was used to determine significant differences (P < 0.05) between the treatments (SAS Inst. Inc., Cary, NC, USA). The results are presented as means and standard errors.

## Results

To better understand the cinnamon effect on the performance of quails in the posture phase, we measured the relative weight of intern organs, egg production parameters and blood parameter related to the lipid metabolism. We also measured the expression of genes related to the antioxidant activity, and oxidative stress markers to evaluate the cinnamon effect on the antioxidant capacity.

### Egg production

For the egg production characteristics evaluated, a significant difference in feed conversion was observed by egg mass (P = 0.0126); the birds of the CPS treatment presented a lower FC/MASS value (2.426 g/g) than those of the control group (2.493 g/g). There was no effect of the treatments on the other evaluated characteristics (Table 3).

**Table 3:** Characteristics related to the production of eggs of laying quail receiving diet no cinnamon supplementation (NCS) and with cinnamon powder supplementation (CPS).

	Average ± SE		*P*_*value*_
	NCS	CPS	
EW (g)	10.505 ± 0.090	10.583 ± 0.065	0.4996
YOLK (%)	32.480 ± 1.445	31.426 ± 0.215	0.4876
SHELL (%)	8.735 ± 0.132	8.858 ± 0.059	0.4141
ALBUMEN (%)	59.160 ± 1.511	60.555 ± 0.232	0.3830
POSTURE (%)	93.766 ± 1.340	95.435 ± 0.465	0.2670
FI (g/bird/day)	24.941 ± 0.225	25.028 ± 0.436	0.8634
EM (g)	9.971 ± 0.194	10.170 ± 0.095	0.3822
FC/MASS (g/g)	2.493 ± 0.016	2.426 ± 0.014	0.0126*
FC/DOZEN (g/dozen)	3.836 ± 0.045	3.705 ± 0.044	0.0648

EW–Egg weight; YOLK–Percentage of yolk; SHELL—Percentage of shell; ALBUMEN–Percentage of albumen; POSTURE–Percentage of posture; FI–feed intake; EM–Egg mass; CA/MASSA–Feed conversion per mass of egg; FC/DOZEN–Feed conversion per dozen eggs; The results described in the table are presented as means with their standard errors (SE).

*Significant by the Student’s t test (P<0.05).

### Relative weight

We observed a significant effect of the inclusion of 9g/kg of powdered cinnamon in the diet for laying quails on the relative weight of the pancreas (*P* = 0.0418), intestine (*P* = 0.0209) and ovary (*P* = 0,0389) (Table 4). Birds of the CPS treatment group presented a lower relative weight of pancreas and intestine and higher ovary relative weight than birds of the control treatment. No effect of diet on poultry weight, liver weight, gut length, gizzard weight and abdominal fat was observed.

**Table 4:** Relative weight of organs and abdominal fat of laying quails receiving diet no cinnamon supplementation (NCS) and with cinnamon powder supplementation (CPS).

	Average ± EP		*P*_*value*_
	NCS	CPS	
Bird weight (g)	172.000 ± 5.8310	174.000 ± 3.6740	0.7791
Liver weight (%)	2.184 ± 0.308	1.982 ± 0.253	0.6262
Pancreas weight (%)	0.242 ± 0.010	0.190 ± 0.019	0.0418*
Intestine weight (%)	3.653 **±** 0.256	2.849 **±** 0.113	0.0209*
Intestine lenght (%)	44.278 ± 1.963	42.350 ± 2.169	0.5285
Ovary weight (%)	3.250 **±** 0.211	3.976 **±** 0.205	0.0389*
Gizzard weight (%)	2.488 ± 0.176	2.412 ± 0.094	0.7141
Abdominal fat (%)	0.889 ± 0.165	1.178 ± 0.126	0.2033

The results described in the table are given by means with their standard errors (SE).

*Significant by the Student’s t test (P <0.05).

### Blood parameters

We observed a significant effect of treatments on CK plasma activity (P = 0.0169); Lower activity was observed in the birds of the treatment CPS. Animals from CPS treatment also had higher ALT activity (2.40U/L, *P* = 0.0497), and higher triglyceride content (1516.60 mg/dL, *P* = 0.0207), uric acid (7.40 mg/dL, *P* = 0.0003) and VLDL (300.40 mg/dL, *P* = 0.0252) than birds receiving the NCS treatment diet. No significant difference (*P*>0.05) was observed for cholesterol, creatinine, glucose and AST levels, as described in Table 5.

**Table 5:** Blood parameters related to the lipid metabolism of laying quails receiving diet with and without inclusion of cinnamon powder.

	Average ± SE		*P*_*value*_
	NCS	CPS	
GLUCOSE (mg/dL)	318.80 ± 4.994	318.20 ± 2.922	0.9200
CHOLESTEROL (mg/dL)	235.40 ± 5.826	229.20 ± 4.106	0.4097
URIC AC. (mg/dL)	4.82 ± 0.245	7.40 ± 0.348	0.0003*
TRIGLYCERI (mg/dL)	1286.40 ± 70.627	1516.60 ± 37.742	0.0207*
VLDL (mg/dL)	257.40 ± 63.568	300.40 ± 6.675	0.0252*
CK (U/L)	3529.20 ± 412.140	2241.60 ± 116.270	0.0169*
CRETININE (mg/dL)	0.180 ± 0.020	0.20 ± 0.001	0.3466
AST (U/L)	420.00 ± 4.370	402.20 ± 16.560	0.3291
ALT (U/L)	1.60 ± 0.245	2.40 ± 0.245	0.0497*

CK—Creatine kinase; AST–Aspartate aminotransferase; ALT–Alanine aminotransferase; TRIGLYCERI–Triglycerides; VLDL–Very low density lipoprotein. The results are average with their standard errors (SE).

* Significant by the Student’s t test (P <0.05).

### Catalase activity and TBARS

**Fig 1:**
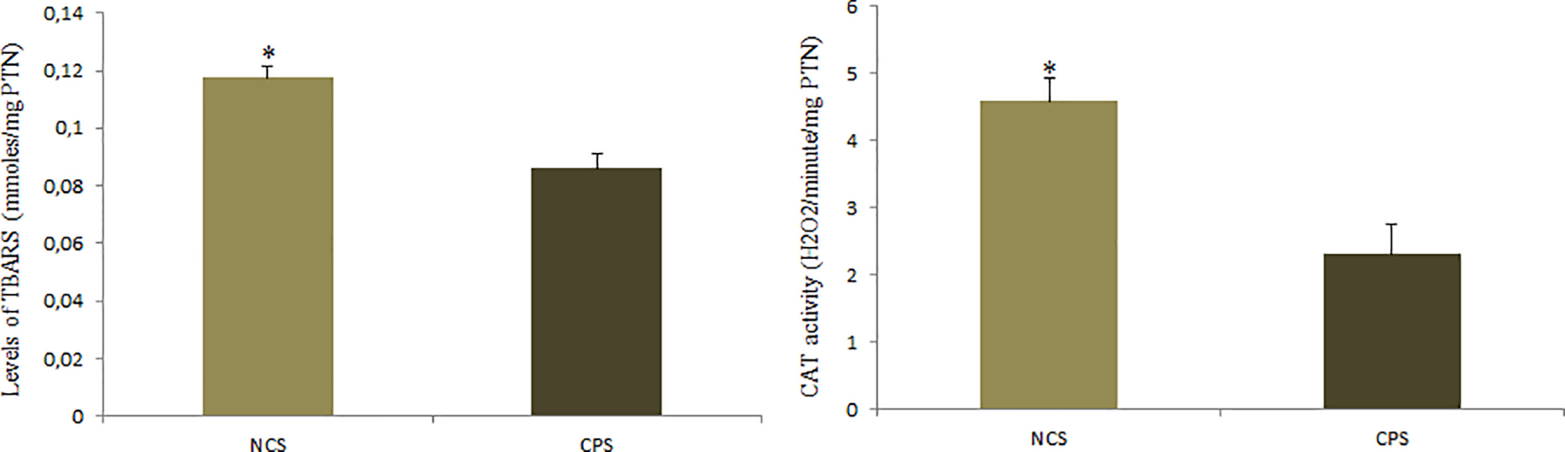
Level of TBARS (nmoles/mg of protein) and CAT enzyme activity (H2O2/minute/mg of protein) in the liver of laying quails receiving diets with (CPS) and without cinnamon powder (NCS). The results are average with their standard errors represented by the vertical bar. * Significant by the Student’s t test (P <0.05).

There was a significant effect of the addition of cinnamon to the diet on the amount of thiobarbituric acid reactive substances (TBARS) (*P* = 0.0158) and on the activity of the enzyme catalase (CAT) (*P* = 0.0033) in the birds’ liver. The birds of the CPS treatment group presented decreased values of TBARS and enzymatic activity; 0.117 nmoles/mg PTN vs. 0.086 nmoles/mg PTN, and 4,585 H_2_O_2_/min/mg PTN vs. 2,304 H_2_O_2_/min/mg PTN) for TBARS and CAT, respectively (Fig 1).

### Gene expression

**Fig 2:**
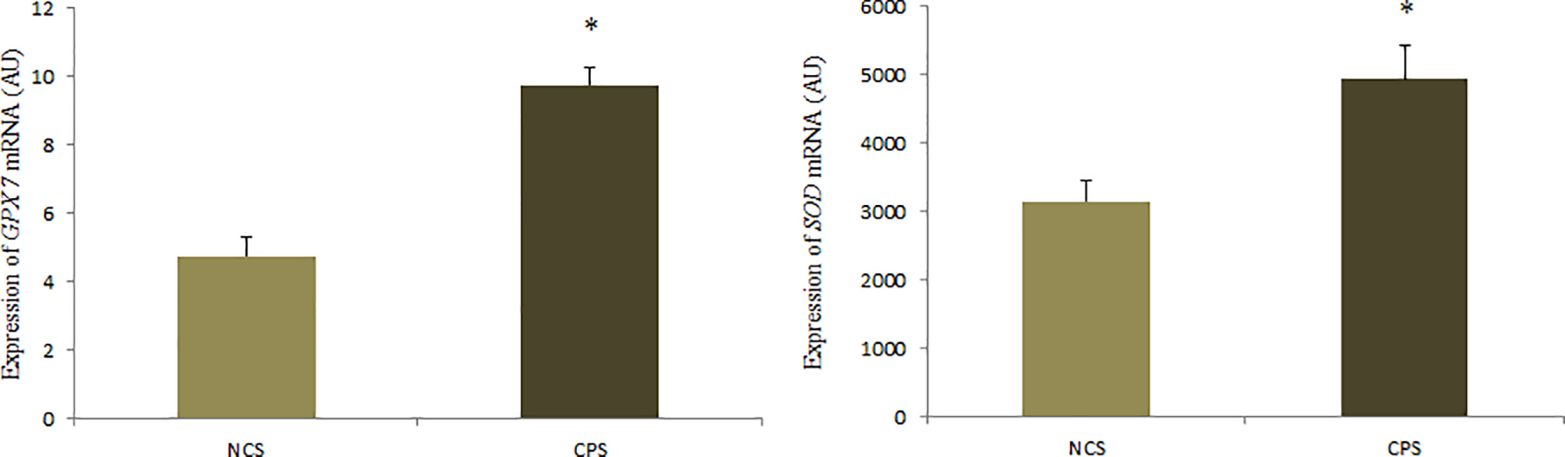
Expression of *SOD* mRNA and *GPx 7* (AU) in the liver of laying quails receiving diets with (CPS) and without cinnamon powder (NCS). The results are average with their standard errors represented by the vertical bar. * Significant by the Student’s t test (P <0.05).

There was a significant difference in *SOD* mRNA expression (P = 0.0074) and *GPx7* (P = 0.0001) between the treatments. In Fig 2, it is possible to observe that the birds fed CPS diet showed higher expression of *SOD* and *GPx7* than animals of the NCS treatment.

**Fig 3:**
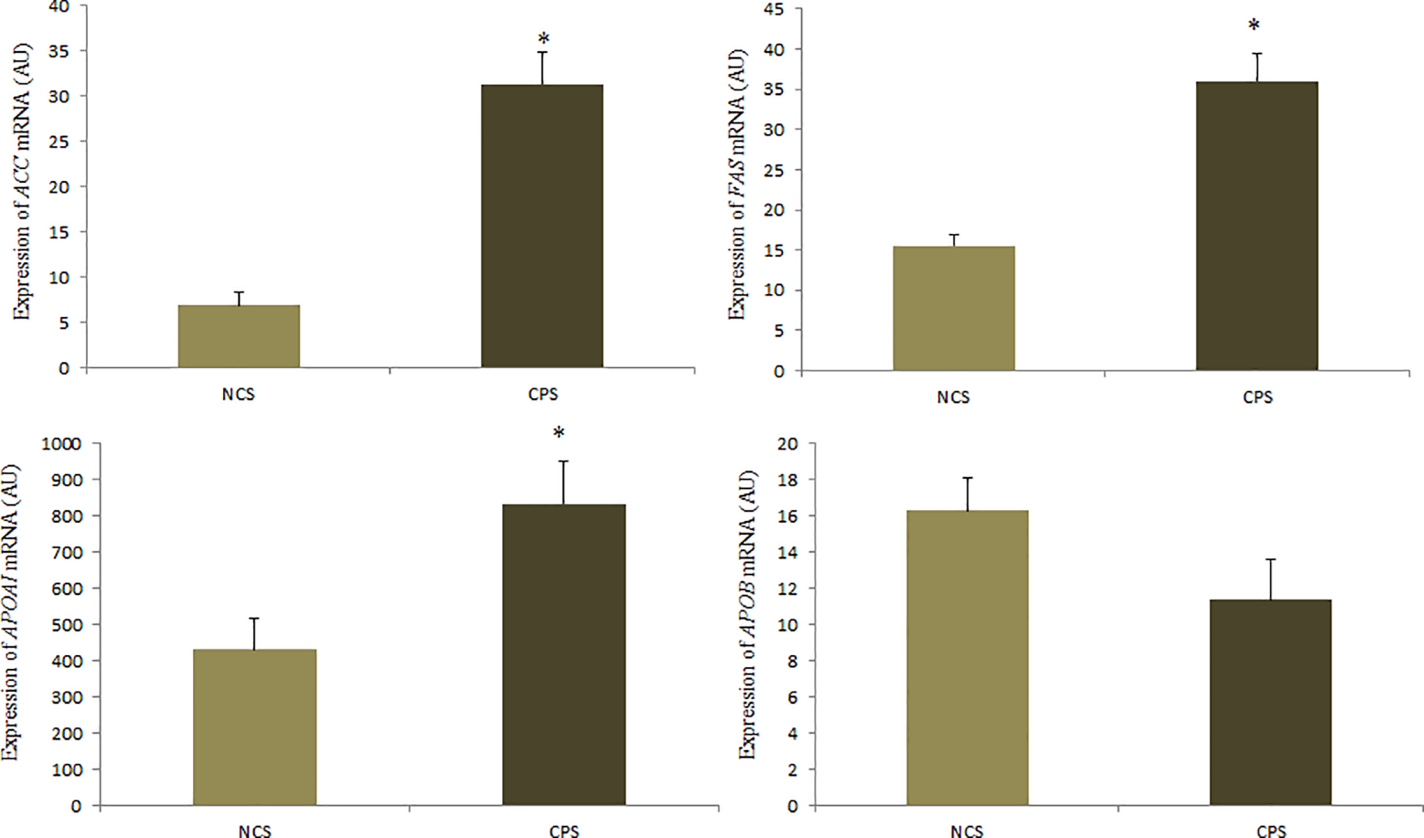
Expression of mRNA *ACC*, *FAS*, *APOA* and *APOB* (AU) in the laying quail’s liver receiving diets with (CPS) and without cinnamon powder (NCS). The results are average with their standard errors represented by the vertical bar. * Significant by the Student’s t test (P <0.05).

A significant difference was observed for *ACC* mRNA expression (P = 0.0001), *FAS* (P = 0.0004) and *APOAI* (P = 0.0159); the expression of these genes was higher in the CPS treatment (31.33, 36.03, and 834.27 AU, respectively), as described in Fig 3. There was no effect of cinnamon addition on *APOB* mRNA expression (P> 0.05).

**Fig 4:**
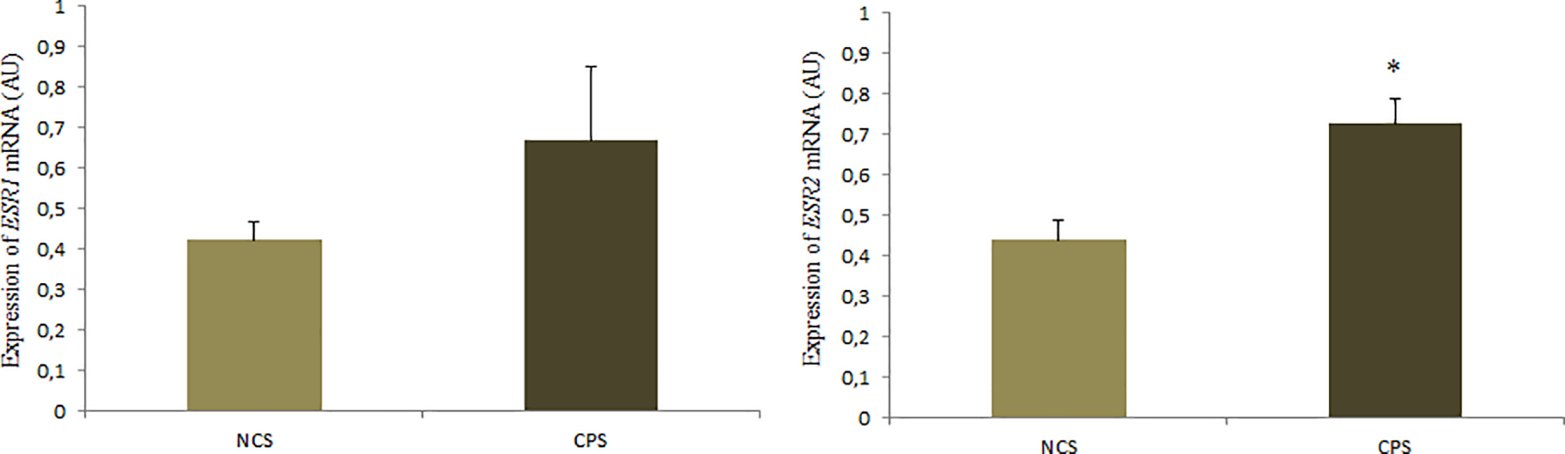
Expression of *ESR1* and *ESR2* (AU) mRNA in the laying quail's liver receiving diets with (CPS) and without cinnamon powder (NCS) added. The results are average with their standard errors represented by the vertical bar. * Significant by the Student’s t test (P <0.05).

The treatments evaluated significantly influenced estrogen receptor 2 gene expression (P = 0.032); birds that received cinnamon inclusion had higher *ESR2* expression than birds that consumed basal diet (0.73 vs. 0.44 AU). There was no significant difference between treatments for *ESR1* gene expression (P = 0.201) (Fig 4).

## Discussion

Quail in the posture phase are more prone to metabolic disturbances and susceptible to free radical damage; this can be justified by the intense metabolism demanded for continuous egg production and environmental stress [1,2]. These factors may adversely affect the proper functioning of the organism, since some metabolic disturbances and the action of free radicals may alter organ morphometry [20] and impair egg production.

ROS are molecules with unstable electrons that can cause degeneration and cell death. These molecules can originate from endogenous or exogenous sources, and when in excess in organisms with metabolic imbalances, can cause damage from the lipid structures of membranes and even the genetic material of cells [21,22]. The antioxidant defense systems rely on the action of substances that have the function of protecting the organism against damage caused by ROS [23]. Among these are enzymatic and non-enzymatic antioxidants, such as eugenol, a compound present in cinnamon, which binds to free radicals by donating a hydrogen molecule, thus promoting the stability of the radicals, and minimizing the damage caused by them [12].

Among the enzymes important for the antioxidant defense system we can highlight superoxide dismutase (SOD), which acts in the control of the production of free radicals, converting superoxide molecules into hydrogen peroxide and oxygen. Hydrogen peroxide (H_2_O_2_) is then eliminated through reactions catalyzed by the enzyme catalase and by the glutathione system that converts H_2_O_2_ to water and molecular oxygen [24]. In this work, it was observed that birds that received the addition of 9g/kg of cinnamon in the diet had higher hepatic expression of the *SOD* and *GPx7* genes than the treatment animals that did not receive supplementation, indicating that cinnamon supplementation may promote an improvement in the antioxidant defense of the organism, not only because it contains compounds that can act directly in the defense against the ROS [12], but also because it modulates the expression of antioxidant enzymes, such as SOD and GPx. Different from that which was expected, the birds of the treatment without cinnamon supplementation presented higher activity of the catalase enzyme (CAT), suggesting that the phenolic compounds present in cinnamon may have different actions on the various enzymes with antioxidant action. According to Rompelberg et al. [11], phenolic compounds such as eugenol mainly influence the activity of enzymes of the glutathione system.

Besides the higher expression of the genes related to the antioxidant capacity, we also observed that birds fed the CPS diet had a lower content of TBARS and higher plasmatic uric acid content than birds fed the NCS diet. Uric acid is considered a potential non-enzymatic antioxidant defense molecule, as it acts as a substrate for ROS oxidation when antioxidant defense systems are not efficient [25,26]; thus, the higher content of uric acid observed in the birds receiving CPS diet may suggest that reduced uric acid content was necessary to act as an electron donor for free radicals because there was greater equilibrium in the neutralization reactions of ROS in CPS birds. On the other hand, in situations where a greater amount of ROS is being produced and there is no higher activity of antioxidant defense systems, a state of oxidative stress can be established in the organism, causing greater damage to macromolecules such as proteins and lipids [27,28]; as a result, the lower amount of TBARS observed in birds fed the cinnamon diet showed that cinnamon exhibited sufficient antioxidant activity to combat ROS produced in the liver of Japanese quails in the posture phase and to ensure metabolic balance.

Besides to TBARS, higher plasma content of CK was also observed in birds receiving the NCS diet. This may indicate that there was more oxidative damage to the muscle proteins of these birds than those treated with cinnamon addition, since elevated levels of serum CK is an indication of changes in cell membrane permeability as a result of tissue damage [29], mainly caused by ROS; membranes damaged by ROS can rupture, causing the cellular contents to extravasate.

Alanine aminotransferase (ALT) and aspartate aminotransferase (AST) are important enzymes for the identification of inflammation and viral infections in the liver, since when detected at high levels in the bloodstream, they may be associated with acute liver diseases [30]; therefore, these are considered physiological markers of some types of damage. In this study, we detected a high level of plasma ALT in animals fed cinnamon; however, according to Georgakouli et al. [30], this cannot be associated with damage caused by free radicals since there is a low correlation between the level of ALT and the damage caused by ROS to liver tissue cells, since these damages are latent and signaling through the circulating ALT level comes from acute damage.

Others manuscripts show the importance of a balance between the production and elimination of free radicals for a good productive performance of birds in posture [31,32,33]. We did not observe any improvement in the percentage of posture due to cinnamon supplementation; however, animals fed cinnamon supplementation presented better conversion by egg mass. This suggests that due to the better metabolic balance achieved with the supplementation of this antioxidant in the diet, there may have been greater mobilization of nutrients for the egg composition of these birds.

The egg has a formation period of approximately 24 hours, which begins soon after ovulation when the yolk is collected by the infundibulum [34]. The yolk precursors are synthesized in the liver in enzymatic reactions that produce the lipid components that will be transported to the ovary via the blood stream in the form of very low density lipoprotein (VLDL). In general, the synthesis of fatty acids begins with the formation of malonyl-CoA with the acetyl-CoA-carboxylase (ACC) enzyme catalyzing this reaction. From this, the multienzymatic fatty acid synthase (FAS) system promotes reactions in successive cycles in which pairs of carbon atoms are coupled to the molecule until the fatty acid is formed [35,36].

The VLDL lipoprotein is essential in the process of transporting the precursors of the egg yolk, since they are of lipid origin, and therefore insoluble in the blood; in order to be transported through the bloodstream, they bind to molecules that allow their transport and recognition in the target tissue, such as apoproteins [2]. The apoproteins A (APOA) and B (APOB) are the two main types, however, they present different functions; the first is associated with the transport of lipids from the tissues to the liver for the production of energy by the oxidation of fatty acids, therefore it is more commonly found in the high density lipoproteins (HDL), while APOB is related to the low and very low density lipoproteins (LDL and VLDL), and it is in the form of these lipoproteins that the transport of triglycerides synthesized in the liver to the target tissues occurs. These are key structures in metabolism, not only because they carry the transport of molecules of lipid origin, but also because they promote the recognition of these molecules in the receptors of cells of target tissue [37].

In our work, we observed that birds fed cinnamon supplementation had greater expression of the *ACC* and *FAS* genes, as well as a higher plasma content of triglycerides and VLDL. This may have been mediated by the action of estradiol (ES2), since a greater expression of this receptor in the liver of the birds that received cinnamon supplementation has been observed, and studies show that E2 produced in large quantities by the pre-ovulatory follicles induce greater expression of genes related to lipid synthesis [38], as well as higher content of VLDL [39]. These results suggest that greater lipid synthesis and greater triglycerides and VLDL content is occurring related to the production of yolk precursors, and may be associated with the better feed conversion by egg mass observed in birds receiving cinnamon in the diet, since it is possible that cinnamon has promoted a greater mobilization of nutrients for the formation of the eggs. The higher production of lipids in laying birds is related to the greater requirement demanded for reproductive organs to be supplied with lipids that will be destined for egg production or for use as precursors of steroid hormones [40,41].

It is possible that disorders of lipid metabolism associated with a greater production of free radicals can cause alterations in the morphometry of important organs. The higher expression of genes related to lipid synthesis has been associated with increased fat deposition [42], and this higher production of fatty acids may be related to the onset of hepatic disorders [1]. In the present study, although we observed greater expression of ACC and FAS genes in birds fed cinnamon, we did not observe a significant effect of diet on visceral fat deposition nor on relative liver weight. These results may be associated with the higher APOA gene expression also observed in these animals, since APOA is involved in the transport of lipids from the peripheral tissues to the liver where they will be metabolized, resulting in the lower deposition of abdominal fat as well as the greater expression of this gene [43].

According to Rodríguez et al. [20], the proper development of the organs implies a better utilization of the nutrients. For laying birds, the productive efficiency may be related to the complete development of the reproductive and digestive systems. In this work, it was observed that cinnamon supplementation increased the weight of the ovary, and reduced the weight of the intestine and pancreas. Since free radicals can decrease the fertility of the birds if the tissues do not have efficient antioxidant mechanisms, the results found in this study may suggest that the greater antioxidant capacity provided by the supplementation of cinnamon was able to maintain the metabolic balance, guaranteeing a better intestinal environment, and favoring the absorption of nutrients, which may have resulted in better development of the reproductive system and making the birds more efficient in producing eggs.

In general, the supplementation of cinnamon powder in the laying quail diet influenced the expression of genes related to antioxidant systems and to lipid metabolism, making egg production more efficient, since it promoted balance in the body in a phase of intense metabolic demand.

## References

[1] Zou XT, Xu ZR, Zhu JL, Fang XJ, Jiang JF. Effects of dietary dihydropyridine supplementation on laying performance and fat metabolism of laying hens. Asian Australasian Journal Of Animal Sciences. 2007; 20(10), 1606.

[2] Wang J, Long C, Zhang H, Zhang Y, Wang H, Yue H, et al Genetic Variant in Flavin-Containing Monooxygenase 3 Alters Lipid Metabolism in Laying Hens in a Diet-Specific Manner. International Journal of Biological Sciences. 2016; 12(11), 1382 doi: 10.7150/ijbs.16472 27877090 10.7150/ijbs.16472 PMC5118784

[3] Bozkurt M, Küçükyilmaz K, Catli AU, Çınar M, Bintaş E, Çöven F. Performance, egg quality, and immune response of laying hens fed diets supplemented with mannan-oligosaccharide or an essential oil mixture under moderate and hot environmental conditions. Poultry science. 2012; 91(6), 1379–1386. doi: 10.3382/ps.2011-02023 22582296 10.3382/ps.2011-02023

[4] Iqbal M, Cawthon D, Wideman RF, Bottje W. Lung mitochondrial dysfunction in pulmonary hypertension syndrome. II. Oxidative stress and inability to improve function with repeated additions of adenosine diphosphate. Poultry Science. 2001; 80(5), 656–665. 11372718 10.1093/ps/80.5.656

[5] Panda AK, Cherian G. Role of vitamin E in counteracting oxidative stress in poultry. The Journal of Poultry Science. 2014; 51(2), 109–117.

[6] Radwan Nadia L, Hassan RA, Qota EM, Fayek HM. Effect of natural antioxidant on oxidative stability of eggs and productive and reproductive performance of laying hens. International Journal of Poultry Science. 2008; 7(2), 134–150.

[7] Gul S, Safdar M. Proximate composition and mineral analysis of cinnamon. Pakistan J Nutr. 2009; n.8, 1456–1460.

[8] Sivapriya T. Cinnamon: bioactive components and usage for thediabetes patients. World Journal of Pharmaceutical Research. 2016; v. 5, n. 3, p. 475–489.

[9] Rao PV, Gan SH. Cinnamon: A Multifaceted Medicinal Plant. Evid Based Complement Alternat Med. 2014; 2014:642942 doi: 10.1155/2014/642942 24817901 10.1155/2014/642942 PMC4003790

[10] Gülçin İ. Antioxidant activity of eugenol: A structure–activity relationship study. Journal of medicinal food. 2011; v. 14, n. 9, p. 975–985. doi: 10.1089/jmf.2010.0197 21554120 10.1089/jmf.2010.0197

[11] Rompelberg CJ, Ploemen JH, Jesperse S, Van Der Greef J.; Verhagen H; Van Bladeren P. J. Inhibition of rat, mouse and human glutation S-transferase by eugenol and its oxidation products. Chem. Biol. Interact. 1996; v. 5, 99(1–3), p. 85–97. 10.1016/0009-2797(95)03662-8 8620581

[12] Şimşek ÜG, Ciftci M, Doğan G, Özçelik M. Antioxidant activity of cinnamon bark oil (Cinnamomum zeylanicum L.) in Japanese quails under thermo neutral and heat stressed conditions. Kafkas Univ Vet Fak Derg. 2013; 19(5), 889–894.

[13] Qotbi AAA. The Effect of Cinnamon Powder and Cinnamon Extract on Performance, Blood Parameters and Microbial Population of Broiler Chicks. Journal of Babylon University/Pure and Applied Sciences. 2016; No.(9)/ Vol.(24).

[14] Mehdipour Z, Afsharmanesh M, Sami M. Effects of dietary synbiotic and cinnamon (Cinnamomum verum) supplementation on growth performance and meat quality in Japanese quail. Livestock Science. 2013; 154, 152–157.

[15] Rostagno HS, et al Brazilian tables for poultry and swine Composition of Feedstuffs and Nutritional Requirements. 3rd ed. Brazil: UFV Viçosa; 2011.

[16] Aebi H. Catalase in vitro. Methods in enzymology. 1984; v. 105, p. 121–126. 6727660 10.1016/s0076-6879(84)05016-3

[17] Bradford MM. A rapid and sensitive method for the quantitation of microgram quantities of protein utilizing the principle of protein-dye binding. Analytical biochemistry. 1976; v. 72, n. 1–2, p. 248–254. 942051 10.1016/0003-2697(76)90527-3

[18] Lei L, Lixian Z. Effect of 24 h fasting on gene expression of AMPK, appetite regulation peptides and lipometabolism related factors in the hypothalamus of broiler chicks. Asian-australas. J. Anim. Sci. 2012; 25(Suppl. 9):1300–1308. doi: 10.5713/ajas.2012.12153 25049694 10.5713/ajas.2012.12153 PMC4092945

[19] Jiang RR, Zhao GP, Zhao JP, Chen JL, Zheng MQ, Liu RR, et al Influence of dietary nicotinic acid supplementation on lipid metabolism and related gene expression in two distinct broiler breeds of female chickens. J Anim Physiol Anim Nutr. 2014; (Berl) 98(Suppl. 5):822–829. doi: 10.1111/jpn.1213 10.1111/jpn.12138 25356484

[20] Rodríguez R, Martinez M, Valdivié M, Cisneros M, Cardenas M, Sarduy L. Morphometry of the gastrointestinal tract and its accessory organs in laying hens fed feedstuffs containing proteinic sugarcane meal. Cuban Journal of Agricultural Science. 2006; 40(3), 343–347.

[21] Freeman BA, Crapo JD. Biology of disease: free radicals and tissue injury. Laboratory investigation; a journal of technical methods and pathology. 1982; 47(5), 412–426. 6290784

[22] Dröge W. Free radicals in the physiological control of cell function. Physiological reviews. 2002; 82(1), 47–95. doi: 10.1152/physrev.00018.2001 11773609 10.1152/physrev.00018.2001

[23] Halliwell B, Gutteridge JMC. Free radicals in biology and medicine. Oxford University Press, USA, 2015.

[24] Fang YZ, Yang S, Wu G. Free radicals, antioxidants, and nutrition. Nutrition. 2002; 18:872–879. http://dx.doi.org/10.1016/S0899-9007(02)00916-4. 12361782 10.1016/s0899-9007(02)00916-4

[25] Machın M, Simoyi MF, Blemings KP, Klandorf H. Increased dietary protein elevates plasma uric acid and is associated with decreased oxidative stress in rapidly-growing broilers. Comparative Biochemistry and Physiology Part B: Biochemistry and Molecular Biology. 2004; 137(3), 383–390. 10.1016/j.cbpc.2004.01.002 15050525

[26] Stinefelt B, Leonard SS, Blemings KP, Shi X, Klandorf H. Free radical scavenging, DNA protection, and inhibition of lipid peroxidation mediated by uric acid. Annals of Clinical & Laboratory Science. 2005; 35(1), 37–45. 15830708

[27] Delles RM, Xiong YL, True AD, Ao T, Dawson KA. Dietary antioxidant supplementation enhances lipid and protein oxidative stability of chicken broiler meat through promotion of antioxidant enzyme activity. Poultry Science. 2014; 93(6), 1561–1570. doi: 10.3382/ps.2013-03682 24879706 10.3382/ps.2013-03682 PMC4988622

[28] Liu CP, Fu J, Xu FP, Wang XS, Li S. The role of heat shock proteins in oxidative stress damage induced by Se deficiency in chicken livers. Biometals. 2015; 28(1), 163–173. doi: 10.1007/s10534-014-9812-x 25503394 10.1007/s10534-014-9812-x

[29] Tabatabaei SM, Badalzadeh R, Mohammadnezhad GR, Balaei R. Effects of Cinnamon extract on biochemical enzymes, TNF-α and NF-κB gene expression levels in liver of broiler chickens inoculated with Escherichia coli. Pesquisa Veterinária Brasileira. 2015; 35(9), 781–787.

[30] Georgakouli K, Manthou E, Fatouros IG, Deli CK, Spandidos DA, Tsatsakis AM. Effects of acute exercise on liver function and blood redox status in heavy drinkers. Experimental and therapeutic medicine. 2015; 10(6). 10.3892/etm.2015.2792 PMC4665762 26668589

[31] Jang I, Ko Y, Kang S, Kim S, Song M, Cho K, et al Effects of Dietary Lutein Sources on Lutein-Enriched Egg Production and Hepatic Antioxidant System in Laying Hens. The Journal of Poultry Science. 2014; 51(1), 58–65.

[32] Iskender H, Yenice G, Dokumacioglu E, Kaynar O, Hayirli A, Kaya A. The Effects of Dietary Flavonoid Supplementation on the Antioxidant Status of Laying Hens. Revista Brasileira de Ciência Avícola. 2016; 18(4), 663–668.

[33] Lin WC, Lee MT, Chang SC, Chang YL, Shih CH, Yu B, et al Effects of mulberry leaves on production performance and the potential modulation of antioxidative status in laying hens. Poultry Science. 2016; pew350. 10.3382/ps/pew350 28339512

[34] Lavelin I, Meiri N, Pines M. New insight in eggshell formation. Poultry Science. 2000; 79(7), 1014–1017. 10901204 10.1093/ps/79.7.1014

[35] Nelson DL, Lehninger AL, Cox MM. Lehninger principles of biochemistry. 5th rev ed. Worth Publishers, New York; 2008.

[36] Poureslami R, Turchini GM, Raes K, Huyghebaert G, De Smet S. Effect of diet, sex and age on fatty acid metabolism in broiler chickens: SFA and MUFA. British journal of nutrition. 2010; 104(02), 204–213. 20199694 10.1017/S0007114510000541

[37] Schneider WJ, Nimpf J, Bujo H. Novel members of the low density lipoprotein receptor superfamily and their potential roles in lipid metabolism. Current opinion in lipidology. 1997; 8(5), 315–319. 9335956 10.1097/00041433-199710000-00011

[38] Choi YI, Ahn HJ, Lee BK, Oh ST, An BK, Kang CW. Nutritional and hormonal induction of fatty liver syndrome and effects of dietary lipotropic factors in egg-type male chicks. Asian-Australasian journal of animal sciences. 2012; 25(8), 1145–1152. doi: 10.5713/ajas.2011.11418 25049674 10.5713/ajas.2011.11418 PMC4092996

[39] Wang XJ, Li Y, Song QQ, Guo YY, Jiao HC, Song ZG, et al Corticosterone regulation of ovarian follicular development is dependent on the energy status of laying hens. Journal of lipid research. 2013; 54(7), 1860–1876. doi: 10.1194/jlr.M036301 23599356 10.1194/jlr.M036301 PMC3679388

[40] Li H, Wang T, Xu C, Wang D, Ren J, Li Y, et al Transcriptome profile of liver at different physiological stages reveals potential mode for lipid metabolism in laying hens. BMC genomics. 2015; 16(1), 763. 26452545 10.1186/s12864-015-1943-0 PMC4600267

[41] Laudadio V, Ceci E, Nahashon SN, Introna M, Lastella NMB, Tufarelli V. Influence of Substituting Dietary Soybean for Air-Classified Sunflower (Helianthus annuus L.) Meal on Egg Production and Steroid Hormones in Early-Phase Laying Hens. Reproduction in domestic animals. 2014; 49(1), 158–163. doi: 10.1111/rda.12245 24134610 10.1111/rda.12245

[42] Xing J, Jjiang Y. Effect of dietary betaine supplementation on mRNA level of lipogenesis genes and on promoter cpg methylation of fatty acid synthase (fas) gene in laying hens. Afr J Biotechnol. 2012; 11: 6633–6640.

[43] Zhuo Z, Lamont SJ, Lee WR, Abasht B. RNA-seq analysis of abdominal fat reveals differences between modern commercial broiler chickens with high and low feed efficiencies. PloS one. 2015; 10(8), e0135810 doi: 10.1371/journal.pone.0135810 26295149 10.1371/journal.pone.0135810 PMC4546421

